# Polygenic risk scores in schizophrenia with clinically significant copy number variants

**DOI:** 10.1111/pcn.12926

**Published:** 2019-09-30

**Authors:** Satoru Taniguchi, Kohei Ninomiya, Itaru Kushima, Takeo Saito, Ayu Shimasaki, Takaya Sakusabe, Yukihide Momozawa, Michiaki Kubo, Yoichiro Kamatani, Norio Ozaki, Masashi Ikeda, Nakao Iwata

**Affiliations:** ^1^ Department of Psychiatry Fujita Health University School of Medicine Toyoake Japan; ^2^ Department of Psychiatry Nagoya University, Graduate School of Medicine Nagoya Japan; ^3^ Medical Genomics Center Nagoya University Hospital Nagoya Japan; ^4^ Medical Engineering Fujita Health University, School of Medical Sciences Toyoake Japan; ^5^ Laboratory for Genotyping Development RIKEN Center for Integrative Medical Sciences Yokohama Japan; ^6^ RIKEN Center for Integrative Medical Sciences Yokohama Japan; ^7^ Laboratory for Statistical Analysis RIKEN Center for Integrative Medical Sciences Yokohama Japan; ^8^ Center for Genomic Medicine Kyoto University Graduate School of Medicine Kyoto Japan

**Keywords:** genome‐wide association study, Klinefelter syndrome, polygenic risk score, triple X syndrome, velocardiofacial syndrome

## Abstract

**Aims:**

Recent studies have revealed that the interplay between polygenic risk scores (PRS) and large copy number variants (CNV; >500kb) is essential for the etiology of schizophrenia (SCZ). To replicate previous findings, including those for smaller CNV (>10kb), the PRS between SCZ patients with and without CNV were compared.

**Methods:**

The PRS were calculated for 724 patients with SCZ and 1178 healthy controls (HC), genotyped using array‐based comparative genomic hybridization and single nucleotide polymorphisms chips, and comparisons were made between cases and HC, or between subjects with and without ‘clinically significant’ CNV.

**Results:**

First, we replicated the higher PRS in patients with SCZ compared to that in HC (without taking into account the CNV). For clinically significant CNV, as defined by the American College of Medical Genetics (‘pathogenic’ and ‘uncertain clinical significance, likely pathogenic’ CNV), 66 patients with SCZ carried clinically significant CNV, whereas 658 SCZ patients had no such CNV. In the comparison of PRS between cases with/without the CNV, despite no significant difference in PRS, significant enrichment of the well‐established risk CNV (22q11.2 deletion and 47,XXY/47,XXX) was observed in the lowest decile of PRS in SCZ patients with the CNV.

**Conclusion:**

Although the present study failed to replicate the significant difference in PRS between SCZ patients with and without clinically significant CNV, SCZ patients with well‐established risk CNV tended to have a lower PRS. Therefore, we speculate that the CNV in SCZ patients with lower PRS may contain ‘genuine’ risk; PRS is a possible tool for prioritizing clinically significant CNV because the power of the CNV association analysis is limited due to their rarity.

Schizophrenia (SCZ) is a major psychiatric disorder with strong genetic background; several genetic epidemiological studies have suggested that the heritability of this disorder is estimated at ~80%^1^; therefore, genetic association studies are considered one of the best tools to detect the susceptibility factors for SCZ. However, no robust evidence for the susceptibility genes with high significance had emerged until the genome‐wide association study (GWAS) was established as the main methodology in genetic studies. Among GWAS results, the Schizophrenia Working Group of the Psychiatric Genomics Consortium (PGC) reported results indicating that 108 loci were associated with SCZ based on the largest sample size.^2^

The most important implication from GWAS results for psychiatric disorders is that the effect size of individual variants (i.e., single nucleotide polymorphisms [SNP]) is small (odds ratio [OR], ~1.1).^2^ In addition, a polygenic model in which the numerous ‘risk’ SNP accumulatively contribute to the development of psychiatric disorders (i.e., SCZ) can help to explain the genetic architecture of such disorders. Specifically, based on this concept, polygenic risk score (PRS) analysis has revealed that subjects with scores within the highest decile have a higher OR (~8–21) for SCZ compared with those with scores within the lowest decile.^2^

In terms of focusing on variants with a large effect size, the most notable are copy number variants (CNV).^3^ As large deletions or duplications can be detected using microarray SNP chip data with sufficient probe density, theoretically, all samples analyzed in GWAS can be examined for CNV analysis, and thus, the PGC CNV group has reported robust results based on a large sample size (21 094 case subjects with SCZ and 20 227 control subjects).^4^ From this result, several large CNV, including the 22q11.2 deletion, 16p11.2 (proximal) duplication, 2p16.3 (*NRXN1*) deletion, 15q13.3 deletion, 1q21.1 deletion/duplication, 3q29 deletion, 16p11.2 (distal) deletion, and 7q11.23 duplication, have been reported as being strong candidates with a large effect size and an OR of ~4–70.^4^

However, it is true that exact CNV calling based on the SNP chip data is limited to sufficiently large CNV (i.e., >20–50kb minimum); therefore, a different methodology, such as array comparative genomic hybridization (aCGH), is an appropriate method to estimate the precise genetic contribution of smaller CNV (i.e., 10kb minimum). Previous studies have reported using this method, and the one with the largest sample size revealed an enrichment of ‘clinically significant’ CNV in subjects with SCZ.^5^ These suggested that ‘small’ CNV contributed to the susceptibility to SCZ in addition to large CNV.

Despite clear evidence of individual susceptible SNP and risk CNV, limited contribution has been explained for developing SCZ so far. Therefore, taking into account the complex architecture underlying the pathophysiology of SCZ, joint analysis combining SNP‐based association analysis or PRS from common variants and CNV analysis for rare variants, including smaller CNV, is essential. From this viewpoint, although the CNV analyzed were large, the PGC CNV group indicated that subjects with ‘large’ CNV (>500kb) tended to have lower PRS, whereas subjects without the ‘large’ (and possibly pathogenic) CNV tended to have higher PRS.^6^ This implicated that CNV in subjects with lower PRS may be prioritized as ‘genuine’ risk CNV.

The present study aimed to examine the joint contribution of PRS and CNV (>10kb) in subjects genotyped using aCGH, as previously reported.^5^ As a working hypothesis, it was suggested that clinically significant CNV, even with a smaller size, may have a substantial impact on SCZ, and thus have lower PRS, similar to the PGC report targeting large CNV.

## Methods

### Subjects

The subjects comprised 737 patients with SCZ and 1227 healthy controls (HC) who were genotyped both using aCGH (720K Whole‐Genome Tiling array, Roche NimbleGen, Madison, WI, USA; and SurePrint G3 Human CGH 400k, Agilent, Santa Clara, CA, USA) and an SNP array (OmniExpressExome v1, Illumina, San Diego, CA, USA). Diagnosis was based on the DSM‐IV. Written informed consent was obtained from all subjects following thorough explanation of the study. The study was approved by the ethics committees of each participating university.

### CNV analysis

This CNV calling method and its quality control (QC) were as reported previously.^5^ In brief, the aCGH analysis was conducted according to the manufacturer's protocols (Roche NimbleGen, Madison, WI, USA and Agilent, Santa Clara, CA, USA). The probe spacing was ~2.5kb, thus sufficient to call ‘smaller’ CNV. The statistical variance of the log2 ratio for each probe was then calculated. For sample‐wise QC, subjects were removed with a QC score >0.15 and with >80 or 45 CNV for NimbleGen and Agilent chips, respectively. Subsequently, for CNV‐wise QC, smaller CNV (<10kb), CNV with a low probe density (<1 probe/15kb), overlapped CNV with segmental duplication (>70%), and CNV in the Y chromosome, with the exception of XXY, were removed. Finally, common CNV (>1%) were filtered for the final analysis.

The definition used for a clinically significant CNV was the same as that used in the previous study,^5^ which is in accord with the guideline based on the American College of Medical Genetics (ACMG): clinically significant CNV involves ‘pathogenic’ CNV and ‘uncertain clinical significance, likely pathogenic.’^7^

### SNP analysis

The samples were part of the previous study^8^; however, QC was performed again in the present study and samples with a low call rate (<0.95) and within two degrees of relatedness (using identity‐by‐state analysis: Pi hat >0.1) were removed for the sample‐wise QC, as were SNP with a large deviation from the Hardy–Weinberg equilibrium (*P* < 10^−6^) and a low call rate (<0.95). Finally, SNP with minor allele frequency >1% were filtered and population stratification was examined by principal component analysis (PCA; Fig. S1), resulting in 1902 samples (724 patients with SCZ and 1178 HC) and 543 339 SNP eligible for subsequent analysis.

### PRS analysis

PRS was calculated using PRSice v1.25 software.^9^ As the discovery statistics to define the ‘risk’ allele for SCZ, we used the results from the meta‐analysis (fixed effect model) between PGC/Chinese samples^10^ and our Japanese samples (following removal of the samples used in this study: total 42 541 cases and 69 191 HC) of the SNP in autosomes.^8^ To prune the SNP in linkage disequilibrium (LD), LD clumping (*r* ^2^ threshold 0.1) was performed and the SNP in the major histocompatibility complex region (chr6: 26–33 Mb) were removed in accord with the previous study.^6^ Consequently, the PRS for 724 SCZ subjects and 1178 HC were calculated based on 10 *P*‐thresholds (Pt: 5 × 10^−8^, 1 × 10^−6^, 1 × 10^−4^, 0.001, 0.01, 0.05, 0.1, 0.2, 0.5, and 1). Furthermore, to generalize the PRS, PCA was performed based on the PRS calculated from the 10 Pts and the first principal component score (PRS1: 62.5% of the variance was explained) was used following the previous study.^6^ The difference of the PRS1 was calculated using the Wald *t*‐test and the significance level was set as 0.05.

## Results

**Figure 1:**
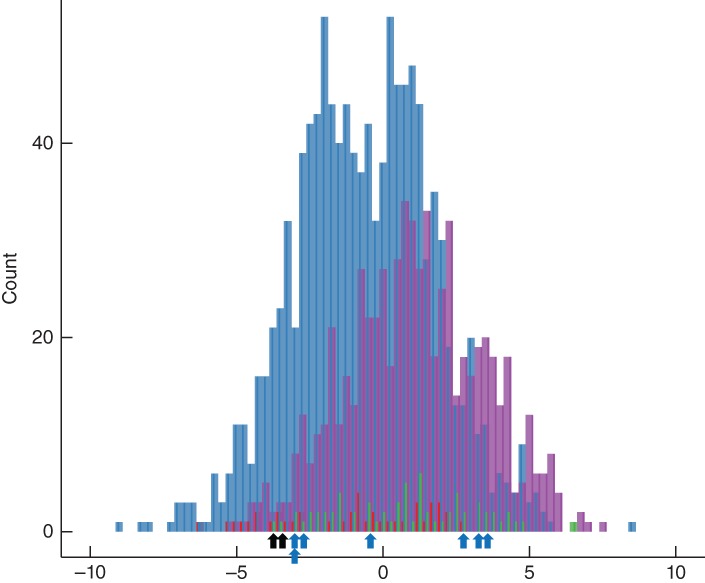
Histogram of the polygenic risk score distribution in subjects with schizophrenia (SCZ) and healthy controls (HC) with/without clinically significant copy number variant (CNV). The *X*‐axis indicates the polygenic risk score based on principal component analysis. (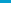) Control with CNV. (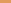) Control without CNV. (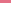) SCZ with CNV. (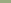) SCZ without CNV. (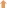) 22q11.2 deletion syndrome. (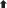) chrX abnormality.

Using our previous whole‐genome SNP datasets, PRS based on the PCA (PRS1) in 724 patients with SCZ and 1178 HC were calculated. First, to confirm the known findings, where PRS in the ‘target’ SCZ samples (based on discovery ‘schizophrenia’ GWAS) was higher than that in the HC subjects,^8^ PRS1 was compared between all cases and all HC without taking CNV into account. The results of this analysis were as expected, with significant difference even in the small sample size (*P* = < 2.2 × 10^−16^; Table 1 and Fig. 1).

**Table 1:** Mean polygenic risk scores based on the principal component analysis (PRS1) of subjects with and without ‘clinically significant’ CNV

Subjects		‘Clinically significant’ CNV	Number of subjects	Mean PRS1	SE	*P*‐value
Schizophrenia	All		724	1.06	0.0861	< 2.2×10^‐16^ †
		No	658	1.09	0.0901	0.283‡
		Yes	66	0.761	0.291	
HC	All		1178	−0.652	0.0694	
		No	1136	−0.640	0.0706	0.427§
		Yes	42	−0.957	0.389	

† Comparison of PRS1 between all cases and all HC (Wald *t*‐test).

‡ Comparison of PRS1 between cases with and without clinically significant CNV (Wald *t*‐test).

§ Comparison of PRS1 between HC with and without clinically significant CNV (Wald *t*‐test).

CNV, copy number variant; HC, healthy controls; PRS1, polygenic risk score 1; SE, standard error.

Subsequently, polygenic contribution was examined in the case‐only analysis subdivided by with/without clinically significant CNV: 66 subjects carried the clinically significant CNV (as defined by the ACMG, including small CNV), whereas 658 subjects had no such CNV. In this comparison, no significant difference in PRS1 was observed between cases with and without the CNV (*P* = 0.283, Table 1).

Additionally, we conducted other comparisons, detecting no differences in PRS1 between HC with and without the CNV (*P* = 0.427, Table 1), but confirming significant differences between: (i) cases with the CNV and all HC (*P* = 1.17 × 10^−5^); (ii) cases with the CNV and HC with the CNV (*P* = 2.80 × 10^−4^); and (iii) cases with the CNV and HC without the CNV (*P* = 1.16 × 10^−5^). It is of note that the direction of the effects in all comparisons was identical to those reported previously.^6^

When focusing on SCZ with clinically significant CNV in the lowest decile (six subjects) of the PRS1 (Table S1), five of the six subjects carried well‐established CNV, three subjects had the 22q11.2 deletion, and two subjects had X chromosome aneuploidies (47,XXY and 47,XXX), although the remaining last one was not major (small deletion in *HECW2*: HECT, C2, and WW domain containing E3 ubiquitin protein ligase 2). Regarding 22q11.2 deletion syndrome, seven of the 66 cases with the clinically significant CNV had this CNV; therefore, in the lowest decile (*N* = 3), significant enrichment of the 22q11.2 deletion syndrome was observed (3/7 out of 66 cases with the clinically significant CNV: permutation *P* = 0.013). Similarly, for the X chromosome aneuploidies, significant enrichment of the CNV was detected in the lowest decile (two cases had the aneuploidies and the PRS1 of them were located in the lowest decile: 2/2 out of 66 cases with the clinically significant CNV: permutation *P* = 0.0069). A total of five subjects with the 22q11.2 deletion and X chromosome aneuploidies were located in the lowest decile (in total, nine subjects had specific clinically significant CNV of all 66 cases with the clinically significant CNV: permutation *P* = 7.9 × 10^−5^).

However, the PRS for subjects with other ‘well‐established’ significant CNV reported in the previous study^4^ (16p11.2 [proximal] duplication, *NRXN1* deletion, 3q29 deletion, and 1q21.1 duplication) or those with smaller clinically significant CNV were distributed randomly.

Whereas, in the clinically significant CNV list for the HC subjects (Table S2), there was no well‐established CNV, with the exception of 16p11.2 (proximal) duplication (the 11th lowest of 42 samples) and *NRXN1* deletion (the 25th lowest of 42 samples), no enrichment was observed in the lowest decile of PRS1.

## Discussion

In the present study, we detected no difference in the PRS between SCZ subjects with and without clinically significant CNV, but a significant difference between cases with the CNV and HC (with/without the CNV). In these comparisons, it is stressed that the expected direction of the effects (e.g., a lower PRS in SCZ subjects with the clinically significant CNV than those without the CNV) was observed in a Japanese population, which is different to that reported previously^6^ (i.e., mainly European ancestry). It was also of note that the specific ‘well‐established’ risk CNV (22q11.2 deletion and chromosome X aneuploidies) were enriched in the lower decile of the PRS in those cases with clinically significant CNV.

In the PRS analysis, we could not detect a significant difference in PRS between subjects with and without clinically significant CNV, which was one of the main results in the previous study^6^; thus, we could not replicate the previous results in which subjects with the CNV had significantly lower PRS compared with those without the CNV.^6^ Furthermore, the present study found that the smaller clinically significant CNV (10–50kb) did not have an ‘overall’ effect on the PRS difference in cases with (mean PRS1 = −1.30) and without (mean PRS1 = −0.14) the CNV (*P* = 0.38).

The small sample size is an obvious limitation; thus, the statistical power is not sufficient to conclude an interplay between clinically significant CNV and PRS. Indeed, the power analysis revealed that the sample size in the present study had only 6% if the PRS1 mean and standard deviation detected in the previous study were used.^6^ However, if our result is not ‘false negative,’ SCZ subjects with clinically significant CNV may also have polygenic contribution based on the common SNP (e.g. PRS), similar to those without the CNV. Such observations (i.e., joint contribution of rare CNV or single nucleotide variant [SNV] and polygenic risk) have been previously reported, although the psychiatric phenotype was different: Niemi *et al*.^11^ reported contribution of common variants to rare severe neurodevelopmental disorders both with and without a diagnostic variant. Weiner *et al*.^12^ similarly found that polygenic variation additively contributes to risk in autism spectrum disorder cases who carried a likely pathogenic de novo variant.

However, in general, some of the clinically significant CNV may contain false positive as a risk, but a small proportion of them reveals ‘genuine’ risk because the clinically significant CNV (and even if the ‘CNV size [e.g., >500kb]’ is used as the definition for the ‘clinically significant’ as first ‘filter’) are not evidently perfect. Thus, it is likely that such joint contribution may influence the development of SCZ subjects who harbor the clinically significant CNV that are not ‘genuinely’ associated with SCZ.

In any situation, the present study highlighted that the enrichment of well‐established ‘large’ CNV (22q11.2 deletion and 47,XXY/47,XXX^4^, ^5^, ^13^) in the lower PRS distribution is suggestive, especially for the prioritizing of clinically significant CNV with higher probability as risk; this is because the sample size in the CNV association analysis did not provide sufficient power to define ‘statistically significant’ CNV because of its rarity (i.e., only eight CNV^4^ were significant even in the large sample size: ~40 000 subjects in total). Therefore, we inductively consider that the prioritization of risk CNV using PRS distribution (in addition to the ACMG definition as first filter) is one of the most useful tools, with CNV in subjects with a lower PRS considered to be a more likely candidate with ‘genuine’ risk CNV or genes. This speculation is partially supported by the same analysis using other traits as ‘discovery’ statistics, in which the significant enrichment was observed only when SCZ GWAS was used for discovery, and not when PRS was calculated using other discovery statistics (e.g., AST quantitative trait analysis,^14^ type II diabetes,^15^ body mass index,^16^ major depressive disorder^17^ and bipolar disorder,^18^ all from Asian ancestry; Fig. S2 and Table S3).

In this regard, *HECW2* deletion, which was located in the lowest decile of the PRS and smaller size (=13kb), is a potent ‘genuine’ risk CNV with higher probability. Recent genetic studies, including whole exome sequencing,^19^, ^20^, ^21^ have shown that de novo mutations in *HECW2* were associated with intellectual disability, which shares pathogenicity with SCZ and autistic spectrum disorder, specifically in relation to the rare variants. The function of *HECW2* in connection with SCZ remains unclear; however, several studies have suggested that *HECW2* acts on a diverse group of proteins, including p73, as a crucial mediator of neurodevelopment and neurogenesis.^22^

Furthermore, it is of interest that the PRS distribution of SCZ patients with 22q11.2 deletion and chromosome X aneuploidies appears to show a ‘negative correlation’ (i.e., PRS in the subjects with such CNV may be protective against the development of SCZ in terms of the polygenic model); however, it is not possible to confirm this assumption as no HC subjects had such CNV, thus further analysis is essential.

For other ‘well‐established’ risk CNV showing significant association in the previous study,^4^ the PRS distributions in the present study were random; therefore, it is not compatible with the assumption based on the above results for 22q11.2 deletion and X chromosome aneuploidies. However, because the number of the subjects with these CNV was limited (*n* = 1–2), we cannot exclude the possibility of false negatives in the lowest decile. In addition, because an additive effect between the clinically significant (despite such CNV containing false positive as a risk, as mentioned above) CNV and PRS is likely for susceptibility for SCZ, it is important to stress that it is not necessary to locate a lower PRS for subjects with such CNV (i.e., a couple of the subjects with 22q11.2 deletion had higher PRS).

Another obvious limitation in the present study was that we did not correct multiple testing as these statistics were not completely independent; thus, correction times were difficult to define. However, the enrichment of 22q11.2 deletion and X chromosome aneuploidies in the lowest decile was significant (permutation *P* = 7.9 × 10^−5^), allowing >600 correction times. Further samples are essential to obtain conclusive results.

In conclusion, the present study examined the relation between clinically significant CNV, detected based on aCGH, and PRS in SCZ. It was found that PRS prioritization for the arbitrarily defined clinically significant CNV (e.g., the ACMG definition) is a possible tool for detecting ‘genuine’ risk genes. This assumption is compatible with the hypothesis that the susceptibility for SCZ is not solely explained by common variants with small effect size, but that for a part of subjects being contributed by rare variants with large effect size. Further investigations are required to obtain conclusive results; however, such ‘PRS prioritization’ is applicable for ‘genuine’ risk CNV detection in SCZ without ‘pre‐filtering’ based on the size (>500kb) or function (e.g., the ACMG definition). Therefore, if this speculation is correct, the SCZ subjects who do not harbor ‘large’ or clinically significant CNV, but have the lowest PRS, may have unknown risk CNV or other risk factors, such as rare SNV^23^, ^24^ and epigenetic changes,^25^ despite no statistical significance in the association analysis. Also, such additive interplay may be expandable for explanation of the genetic architecture in SCZ subjects based on joint effect between rare variants, such as that between primary pathogenic variant and rare modifier variants.^26^, ^27^

## Disclosure statement

The authors declare no conflicts of interest.

## Author contributions

S.T., K.N., I.K., T. Saito., A.S., T. Sakusabe., Y.K., and M.I performed the data analysis. S.T., K.N., and M.I. wrote the manuscript. M.K., N.O., and N.I. provided the outlines for the presentation of the study, supervised the study process, and edited the final manuscript. All authors have reviewed the process of the data analysis and writing of the manuscript and approved the final article.

## Supporting information


**Figure S1.** Results of principal component analysis.
**Figure S2.** Histogram of the polygenic risk score distribution in schizophrenia/controls with/without ‘clinically significant’ copy number variants.

**Table S1.** Polygenic risk scores based on the principal component analysis (PRS1) of the subjects with ‘clinically significant’ copy number variants
**Table S2.** Polygenic risk scores based on the principal component analysis (PRS1) of the control subjects with ‘clinically significant’ copy number variants
**Table S3.** Mean polygenic risk scores based on the principal component analysis (PRS1) and enrichment of the subjects with 22q11.2 deletion and X chromosome aneuploidies in the lowest decile of the PRS1
