# Supplementary material for: Polygenic risk scores in schizophrenia with clinically significant copy number variants
Source: Psychiatry Clin Neurosci. 2019 Sep 30;74(1):35–9. doi: 10.1111/pcn.12926 (PMC6973280; doi:10.1111/pcn.12926)

**Supplementary Figure 1: Results of principal component analysis**

Results of the East Asian subjects: GWAS cases and controls and JPT and CHB populations of the HapMap subjects

Case_OUT: cases excluded in the association analysis, Control_OUT: controls excluded in the association analysis, JPT_OUT: HapMap JPT sample distributing outside of the “Mainland of Japan” cluster


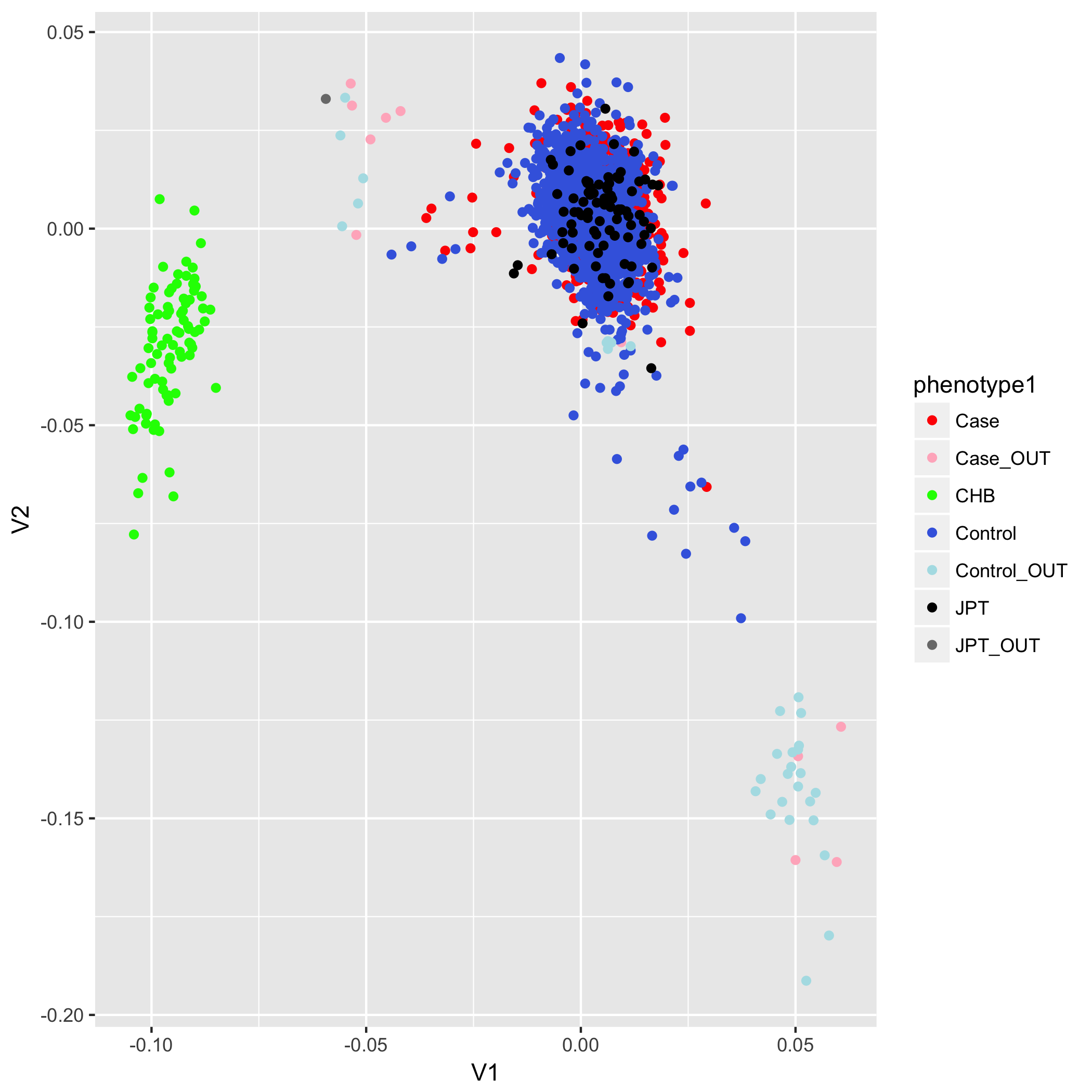


**Supplementary Figure 2: Histogram of the polygenic risk score distribution in schizophrenia/controls with/without “clinically significant” copy number variants**

X axis indicates the polygenic risk score based on the principle component analysis (PRS1).

CNV: Copy number variant, SCZ: schizophrenia

Discovery statistics: (A) schizophrenia, (B) AST, (C) Body Mass Index (BMI), (D) type 2 diabetes, (E) major depressive disorder, (F) Bipolar disorder

1.
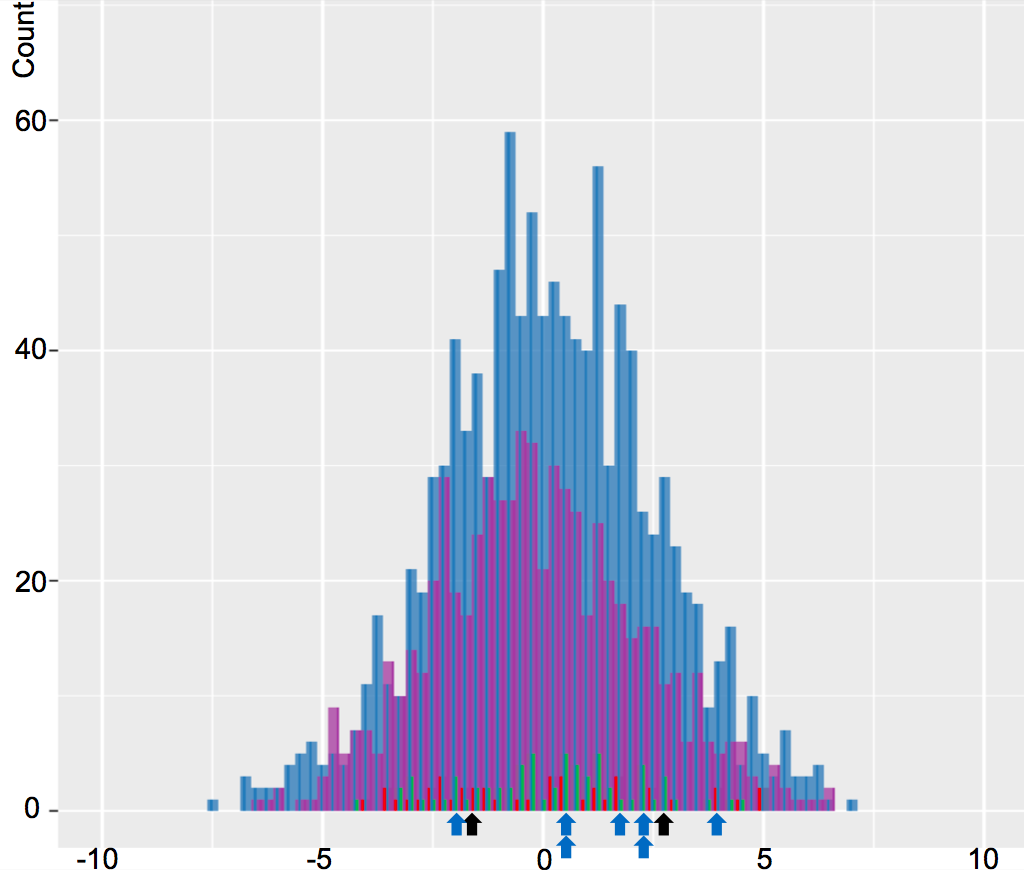

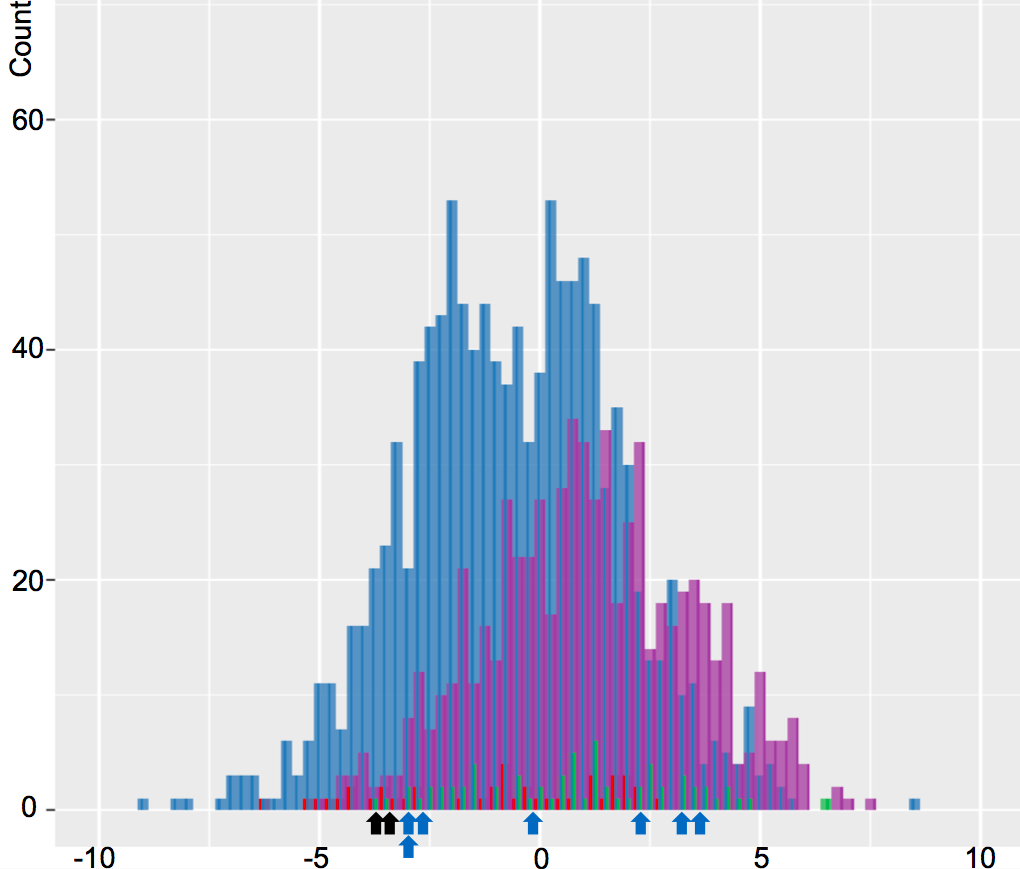

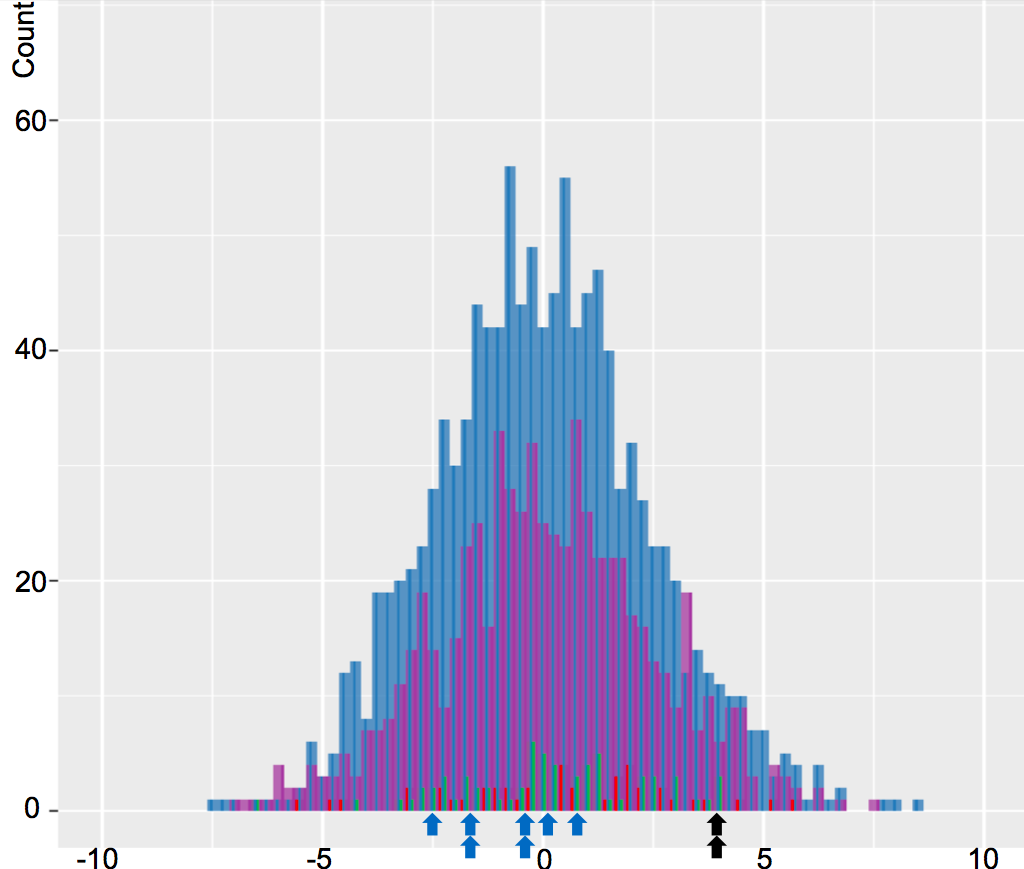
 Discovery: schizophrenia (B) Discovery: AST (C) Discovery: BMI


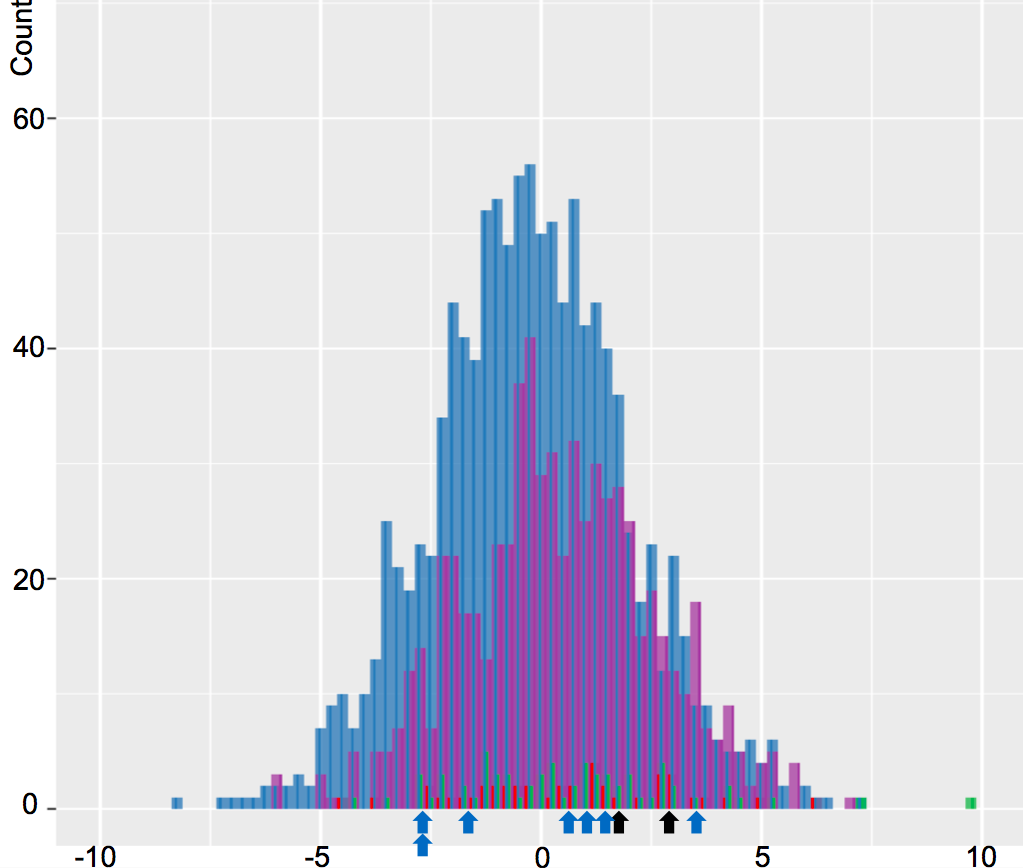

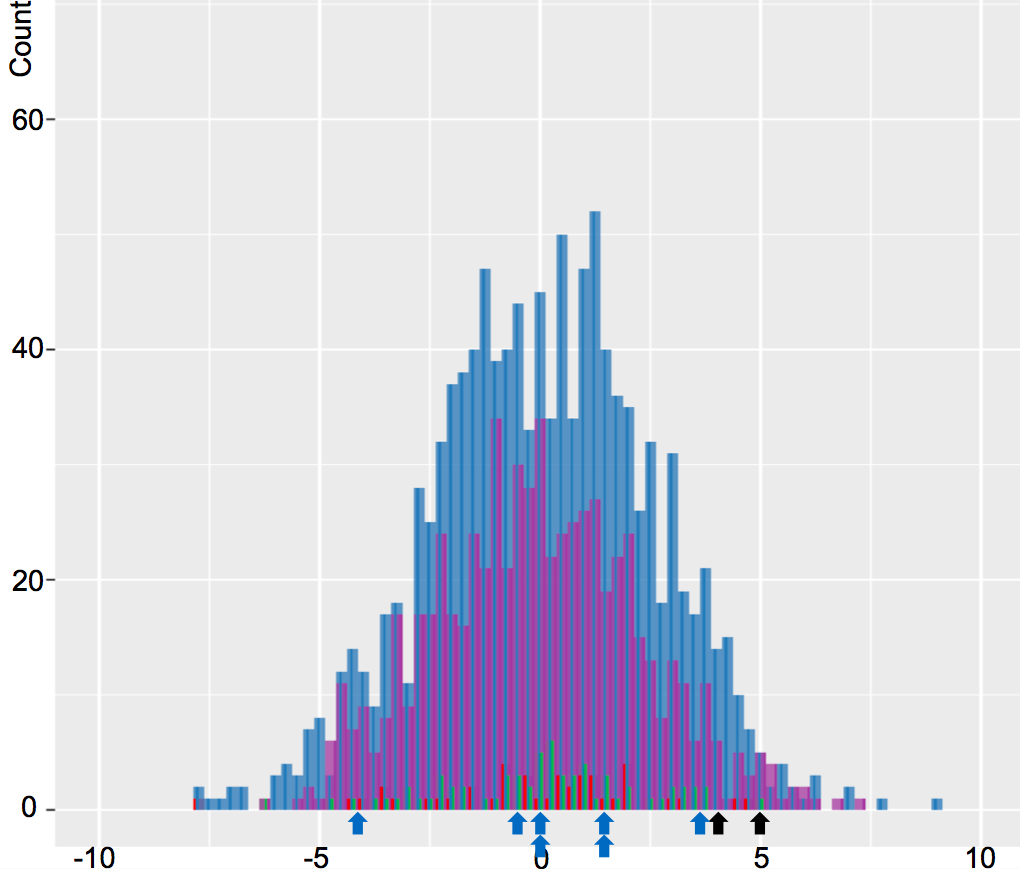

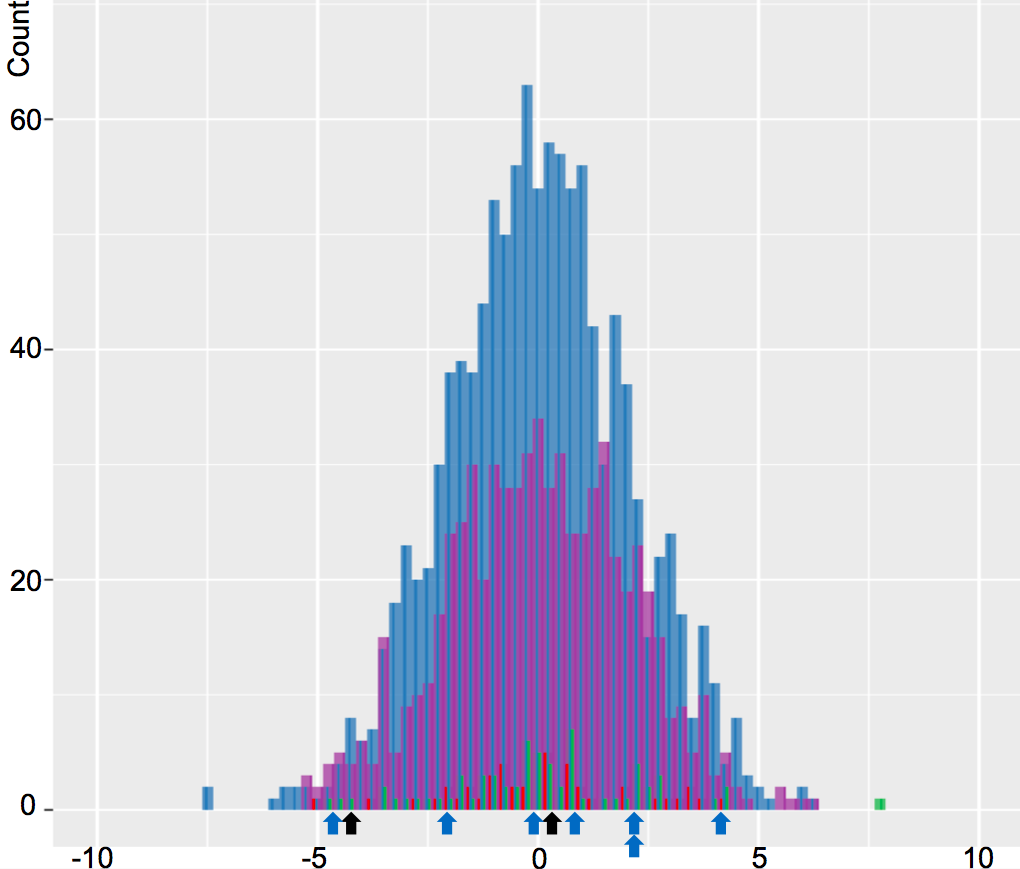
(D) Discovery: type2 diabetes (E) Discovery: Major Depressive (F) Discovery: Bipolar disorder disorder


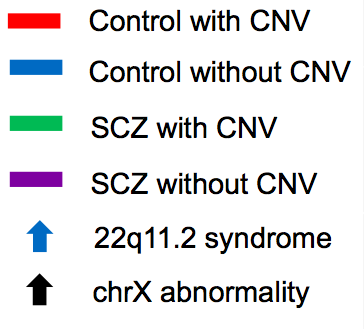

Supplement: Supplementary file 1 — Figure S1. Results of principal component analysis. Figure S2. Histogram of the polygenic risk score distribution in schizophrenia/controls with/without ‘clinically significant’ copy number variants. [file PCN-74-35-s001.docx]
